# Changes in Chopart joint load following tibiotalar arthrodesis: in vitro analysis of 8 cadaver specimen in a dynamic model

**DOI:** 10.1186/1471-2474-8-80

**Published:** 2007-08-08

**Authors:** A Suckel, O Muller, T Herberts, N Wulker

**Affiliations:** 1 Orthopaedic Department, Tubingen University Hospital, Hoppe-Seyler Str. 3, 72076 Tubingen, Germany; 2 Department of Medical Biometry, University of Tubingen, Westbahnhofstrasse 55, 72070 Tubingen, Germany

## Abstract

**Background:**

In the current discussion of surgical treatment of arthroses in the ankle joint, arthrodesis is in competition with artificial joint replacement. Up until now, no valid biomechanical findings have existed on the changes in intraarticular loads following arthrodesis. One argument against tibiotalar arthrodesis is the frequently associated, long-term degeneration of the talonavicular joint, which can be attributed to changes in biomechanical stresses.

**Methods:**

We used a dynamic model to determine the changes in intraarticular forces and peak-pressure in the talonavicular joint and in the calcaneocuboid joint on 8 cadaver feet under stress in a simulated stance phase following tibiotalar arthrodesis.

**Results:**

The change seen after arthrodesis was a tendency of relocation of average force and maximum pressure from the lateral onto the medial column of the foot. The average force increased from native 92 N to 100 N upon arthrodesis in the talonavicular joint and decreased in the calcaneocuboid joint from 54 N to 48 N. The peak pressure increased from native 3.9 MPa to 4.4 MPa in the talonavicular joint and in the calcaneocuboid joint from 3.3 MPa to 3.4 MPa. The increase of force and peak pressure on the talonavicular joint and decrease of force on the calcaneocuboid joint is statistically significant.

**Conclusion:**

The increase in imparted force and peak pressure on the medial column of the foot following tibiotalar arthrodesis, as was demonstrated in a dynamic model, biomechanically explains the clinically observed phenomenon of cartilage degeneration on the medial dorsum of the foot in the long term. As a clinical conclusion from the measurements, it would be desirable to reduce the force imparted on the medial column with displacement onto the lateral forefoot, say by suitable shoe adjustment, in order to achieve a more favourable long-term clinical result.

## Background

The surgical treatment of ankle joint arthrodeses is currently a subject of hot debate, where many authors view arthrodesis as the standard treatment [1-3]. Providing patients with endoprosthetics is a promising alternative and, with the improvement of recent component designs, there are reports of successful long-term outcomes [4] and unexpectedly good results upon return to sporting activity [5]. In the event of a failed endoprosthesis, a problematic situation arises, characterized by significant loss of bone, a high pseudoarthrosis rate and bad clinical results [6]. Thus, the modern prosthetic – with its relative advantage of retention of function and good clinical results accompanied by the potential of an outcome fraught with complications – is in competition with the tried and tested arthrodesis. Here, the reliably attainable lack of pain in the medium-term posed against the threat of long-term joint degeneration in the subtalar and transverse hindfoot joint ranks high on the list of current interests [2,7,8], and until now, there has been no clear evidence of either an alternative treatment or even a less drastic therapy regime. In light of this situation, a biomechanical understanding of the complex hindfoot anatomy is especially important. A possible change in the distribution of forces and peak pressure loads in the Chopart joint has so far not been reliably documented, and it is our intention to investigate these effects after simulated tibiotalar arthrodesis in a realistic dynamic cadaver model and thus to add a biomechanically substantiated basis to the current debate.

## Methods

Eight macroscopically and roentgenographically normal foot specimens were tested comparing tibiotalar arthodesis vs. nativ situation on a dynamic gait simulator. The stance-phase of walking was simulated from heel-contact to toe-off. Ground reaction forces were simulated by a tilting angle- and force-controlled translation stage upon which a pressure measuring platform was mounted (EMED SF1, Novel GmbH, Munich, Germany). Force were applied to the tendons of the foot flexor and extensor muscle groups by cables attached to an additional set of six force-controlled hydraulic cylinders (GHS GmbH, Ilsfeld-Auenstein, Germany). Tibial rotation was produced by an electrical servo motor (Megatorque Motor System, NSK Ltd., Tokyo). The foot simulator functions inversely and was described previously [9]. The pressure measuring platform (50 by 30 cm, 1 sensor/cm^2^) upon which the foot „walked“ was mounted on a hydraulically activated translation platform. The platform was elevated by means of a force-controlled hydraulic cylinder which was programmed and calibrated to simulate the vertical component of the ground reaction force. The translation stage was further allowed to move freely in the medial-lateral, and anterior-posterior directions. The platform was tilted by a second angle-controlled hydraulic cylinder in order to simulate tibial inclination (sagittal plane tibial versus ground angle).

Lower limb specimens were obtained by transection at approximately mid-tibial length. Soft tissues were removed to roughly 4 cm above the ankle joint. The tendons of the lower-leg muscles were blunt dissected free of the leg to allow attachment via clamps to cable pulls by means of which force was applied. Nine tendons of the foot were simulated with six hydraulic cylinders: the triceps surae, tibialis posterior, flexor hallucis longus and flexor digitorum longus combined, tibialis anterior, peroneus longus and brevis combined, and combined extensor digitorum longus and extensor hallucis longus.

The proximal ends of the specimen tibia and fibula were prepared clear to the surface of the bone and potted in their relative anatomical positions in a cold-curing methylmethacrylate resin (Technovit 4071, Heraeus Kulzer, GmbH, Wehrheim, Germany). The potted tibia and fibula were mounted concentrically and vertically oriented in an aluminium tibia mounting cylinder using centring screws. Neutral rotation of the tibia was defined prior to testing by turning the mounting cylinder to orient the foot in 12° of external rotation with respect to the long axis of the pressure measuring platform.

**Figure 1:**
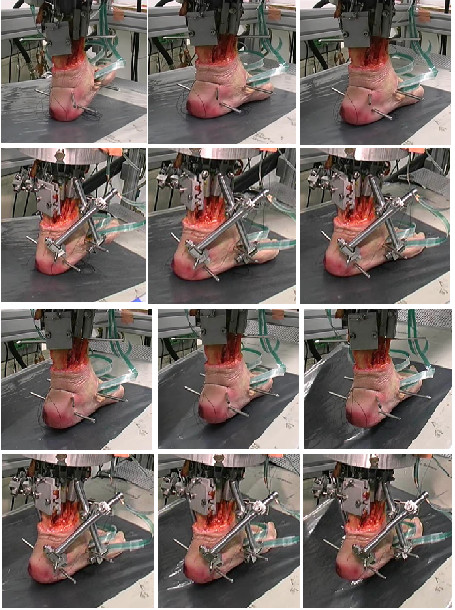
Simulated gait cyclus over full stance of 60 sec. first row: nativ measuring cyclus, cyclus at 5 sec., 15 sec., 25 sec., second row: tibiotalar arthrodesis, cyclus at 5 sec., 15 sec., 25 sec., third row: nativ measuring cyclus, cyclus at 35 sek., 45 sec., 55 sec. fourth row: tibiotalar arthrodesis, cyclus at 35 sek., 45 sec., 55 sec. (additional pin for subtalar arthrodesis still in tuber calcanei)

Physiological gait was simulated from heel strike (0%) to toe-off (100%) over a standardised time of 60 s (fig. 1). Activation patterns for normal gait were used to prescribe forces for each simulated muscle, as was reported before [9]. Based on previous experiments the muscle force for each tendon was set at: Achilles tendon – 780 N at 70% stance phase, tibialis posterior – 225 N at 75%, peroneal tendons – 132 N at 70%, flexor hallucis – 90 N at 85%, tibialis anterior – 66 N at heel contact and extensors of toes – 19 N at 90%. This took into account deviation from normal physiology caused by lack of function of the intrinsic muscles and a wide range of variation in physiologic walking patterns. All specimen were axial loaded with 35 kg to prevent damage of the arthrodesis and to avoid disconnection of the tendon-clamps, this load was tested in previous studies and shown unproblematic for stability of the model.

Intraarticular pressure was measured with resistive pressure sensors (K-scan sensor, map 4201, Tekscan Inc., Boston, MA). The sensor sheets with a thickness of 0.3 mm consists of 264 different sensors arranged in an 11 by 24 matrix with a spatial resolution of 1,8 mm and a maximum pressure range of 2000 PSI (i.e. 13,8 MPa). The pressure values were recorded with the manufactures software (Iscan) with a recording rate of 2 Hz for a time span of 60 seconds to achieve 120 pressure frames. Raw data were then exported as ASCII-Files and further analyzed in MatLab (TheMathwork Inc, Natick, MA).

Separate sheets were used for the talonavicular and the calcaneocuboid joints, with measurements taken simultaneously during the experiment. Therefore the initial sheet was cut along appropriate conducting path to get sensor areas of 9 by 24 mm and 11 by 24 mm respectively that fitted to the anatomic conditions of the joints. The cutted sheets were sealed carefully to avoid wetting and fixed inside the joint with sutures to the capsular tissue. During the experiments, the position was closely followed by the investigators in order to exclude displacement of the sensors during foot motion. Each experiment was performed with five repetitions. The applied load of the joint was characterized by the time course of the force acted upon the sensor area for each pressure frame and the maximum peak pressure value (mpp) within each frame. Mean values for the time course of the 5 repetitions and the 95%-confidence interval were calculated to visualize possible differences in the time course of force and mpp graphically.

Tibiotalar arthrodesis was performed with a stabile fixation using external fixator without removing the cartilage. One pin was placed in the distal tibia and another two pins were placed in the talus body. The pins were connected under maximal compression in a triangle construction medial and lateral. Each specimen was checked for correct placement of the pins using x-ray images and checked for stability under load application prior to start the nativ measuring cycles before fixing the foot in the model, free movement of the neighbouring joints was controlled as well. An additional pin was placed in the tuber calcanei for later study of subtalar arthrodesis. Special concern was taken not to affect tendon-gliding by the Steinmann pins

Measurements were taken first for nativ load for five cycles with the fixateur pins still in situ. After completing the nativ measuring the pins were connected to simulate tibiotalar arthrodesis without changing the simulating conditions to get same conditions for the five arthrodesis runs. Tibial rotation, muscleforce simulation and ground reacting forces were not changed.

The data were analyzed using the statistical software JMP IN 5.1 (SAS Institute Inc., Cary, NC, USA). For each of the 8 feet and each of the 5 replications, the measurements consisted of the values for force and peak pressure for 120 points in time. As dependent variables, we took the mean of the 120 values of the force and the maximum of the 120 peak pressures to get overall values for the whole stance phase. Since these were not normally distributed, we used the Box-Cox transformation of the data to obtain normally distributed data with equal variances. An analysis of variance was performed with the state (native vs. arthrodesis), the joint, their interaction and the length of the foot as fixed factors and the foot number as random factor. Furthermore, an analysis of variance was carried out for each joint separately, now with the state and the length of the foot as fixed factors and the foot number as random factor. For obtaining the median and 95% confidence intervals (95% CI), the means and 95% CIs of the Box-Cox transformations were transformed back.

## Results

**Figure 2:**
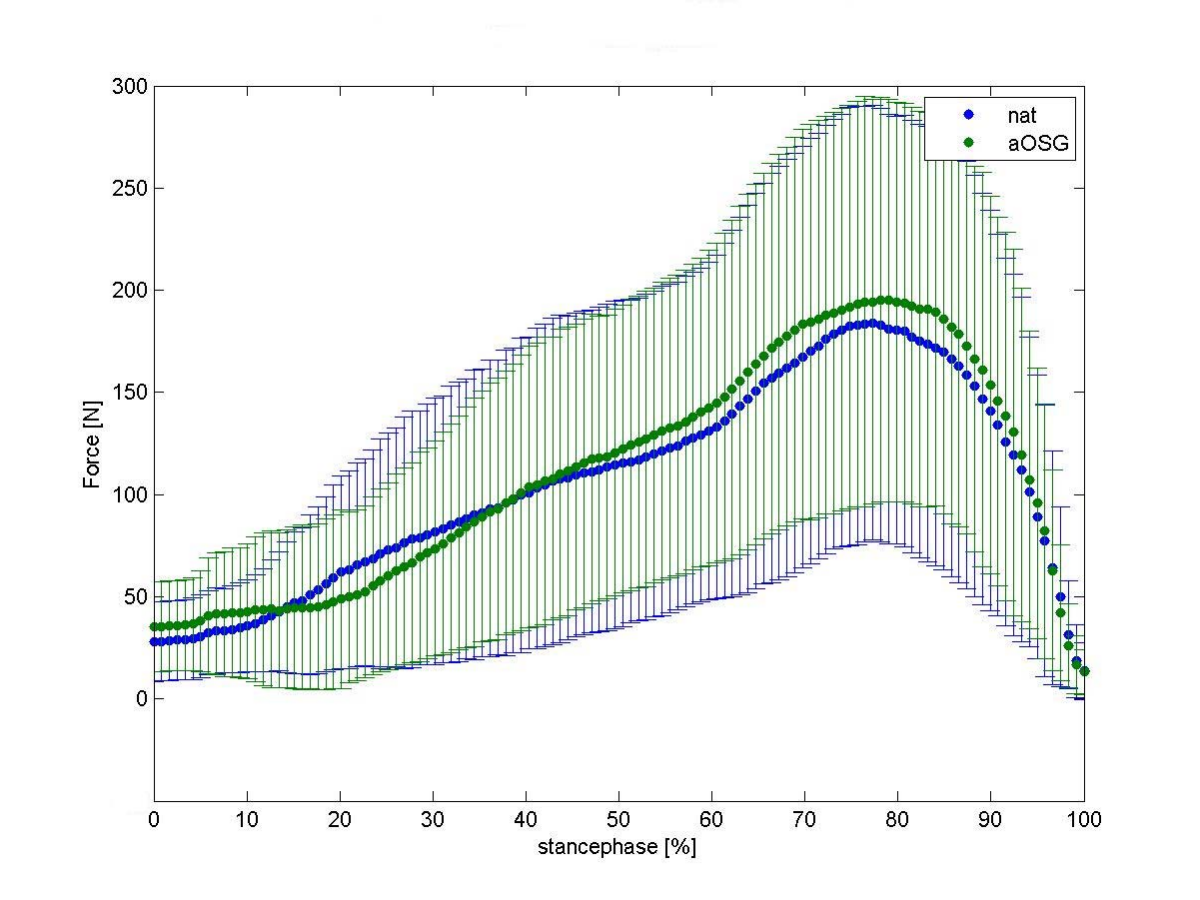
Mean curve of force of 8 foot-specimen, talonavicular joint. summerized diagramm for all feet, mean value and standard deviation. To get this diagram we first averaged the time course of the force values for each foot (i.e. five runs per foot). Each avaraged time course was then normalized to 100% by the respective maximum value of force. The time course for all feet represents therefore the avaraged time course of all the normalized force values. Force values for native (native) and tibiotalar arthrodesis (aOSG) are overlayed as depicted in the legend insert.

**Figure 3:**
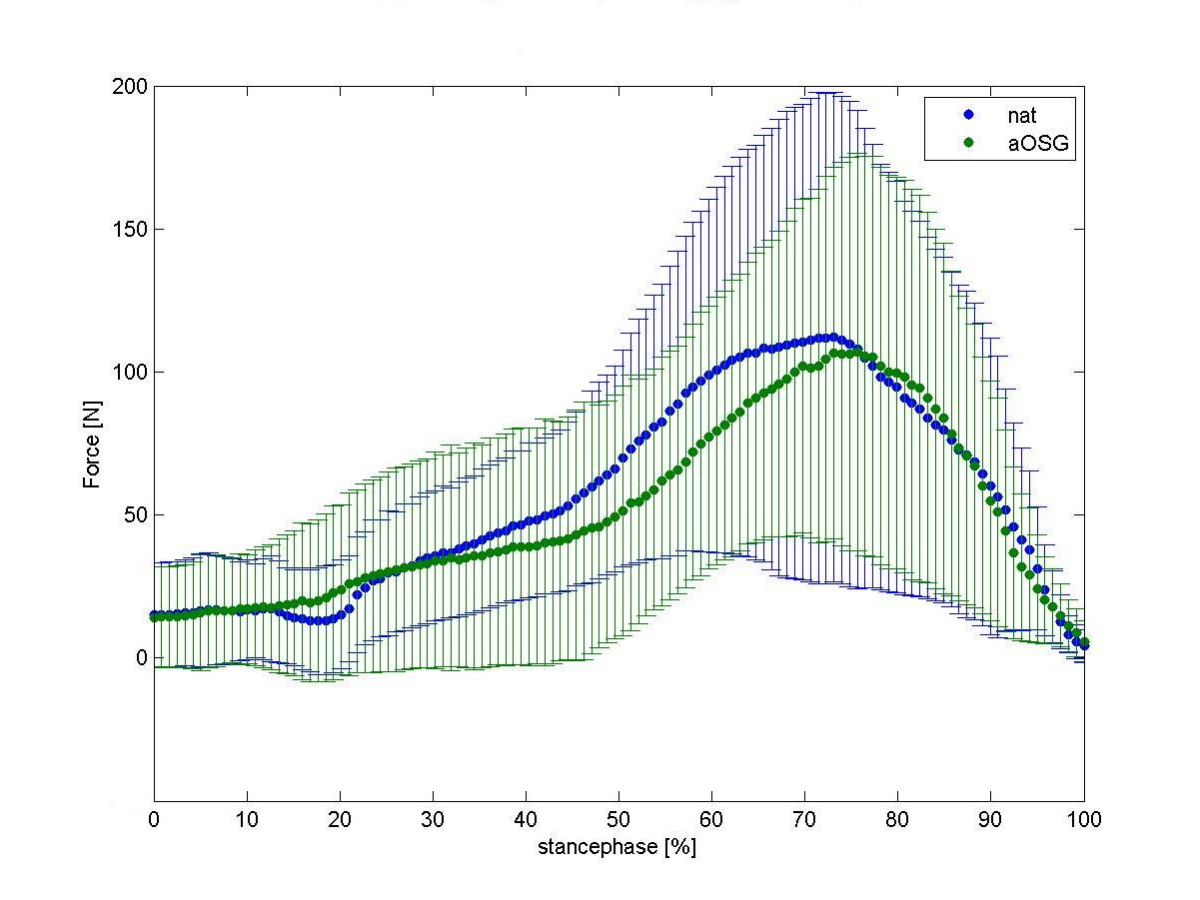
Mean curve of force of 8 foot-specimen, calcaneocuboid joint. summerized diagramm for all feet, see legend fig. 2.

**Figure 4:**
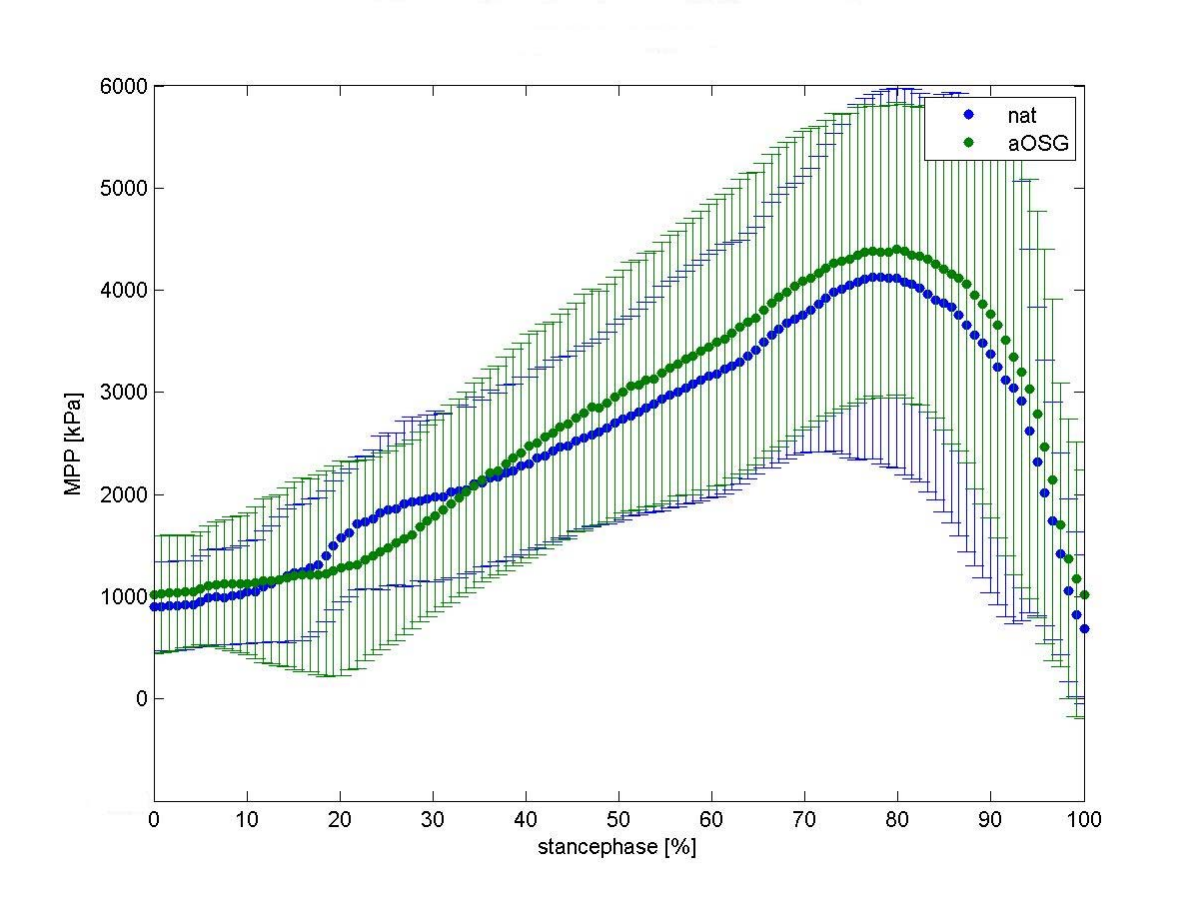
Mean curve maximum peak pressure (mpp) of 8 foot-specimen, talonavicular joint. summerized diagramm for all feet, mean value and standard deviation. To get this diagram we first averaged the time course of the MPP values for each foot (i.e. five runs per foot). Each avaraged time course was then normalized to 100% by the respective maximum value of MPP. The time course for all feet represents therefore the avaraged time course of all the normalized MPP values. MPP values for native (native) and tibiotalar arthrodesis (aOSG) are overlayed as depicted in the legend insert.

**Figure 5:**
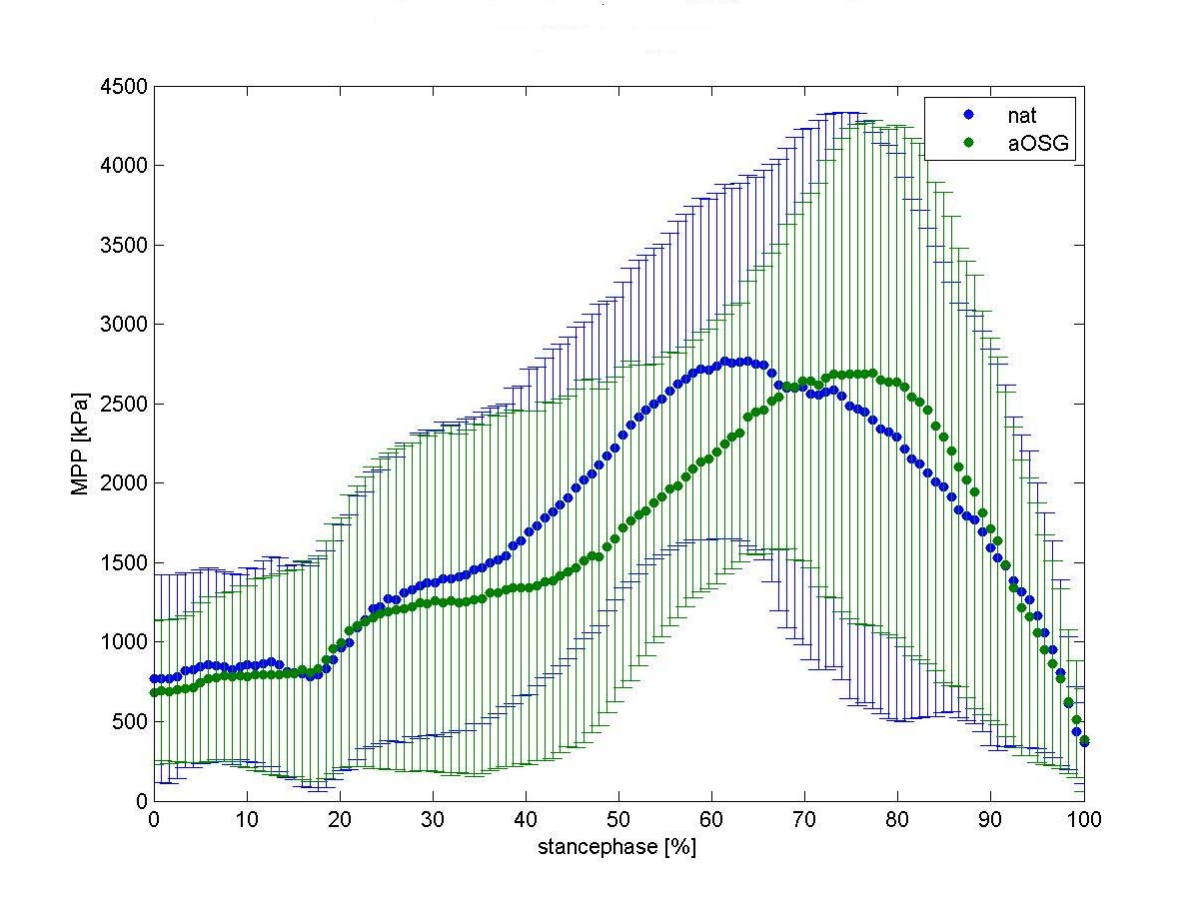
Mean curve maximum peak pressure (mpp) of 8 foot-specimen, calcaneocuboid joint. see legend fig. 4.

We present a mean normalised time course for the force values in the talonavicular joint in figure 2 and in calcaneocuboid joint in figure 3. Differences between native feet and arthrodeses are shown in colour for each moment of the stance phase. The mean time course of maximum peak pressure (MPP) is presented in figure 4 for the talonavicular joint and in figure 5 for the calcaneocuboid joint.

The values for median force during stance phase of both parts of Chopart's joint for 8 foot specimen is given in table 1 as well as for maximum peak pressure in table 2.

**Table 1:** Differences of force transfer in Chopart's-joint

specimen	TN – native	TN – fusion	CC – native	CC – fusion
1	146 (15.4)	89 (5.3)	64 (10.5)	43 (11.8)
2	94 (11.2)	95 (3.6)	8 (1.4)	3 (1.8)
3	70 (5.1)	114 (6.4)	32 (2.0)	71 (3.0)
4	102 (2.0)	184 (22.0)	19 (1.5)	15 (1.4)
5	246 (3.3)	209 (14.0)	97 (7.1)	63 (7.6)
6	42 (1.2)	42 (1.2)	87 (1.9)	55 (2.9)
7	45 (1.1)	58 (4.6)	63 (1.6)	73 (3.6)
8	90 (0.9)	80 (7.0)	75 (3.4)	80 (8.4)
median	92	100	54	48

Results for 8 specimen, mean force of 5 measuring cycles of talonavicular joint (TN) and calcaneocuboid joint (CC), native situation vs. ankle fusion

**Table 2:** Differences of peak-pessure in Chopart's-joint

specimen	TN – native	TN – fusion	CC – native	CC – fusion
1	3.5 (0.2)	3.3 (0.2)	3.8 (0.4)	3.9 (0.7)
2	8.2 (0.1)	7.1 (0.3)	3.1 (0.1)	1.9 (0.6)
3	4.0 (0.1)	5.4 (0.2)	3.4 (0.2)	3.4 (0.2)
4	4.4 (0.1)	6.4 (0.5)	1.3 (0.1)	1.0 (0.4)
5	5.8 (0.2)	6.1 (0.2)	5.9 (0.1)	5.0 (0.1)
6	2.8 (0.1)	3.2 (0.1)	3.1 (0.1)	3.3 (0.1)
7	2.2 (0.2)	3.4 (0.4)	2.3 (0.1)	2.9 (0.2)
8	3.8 (0.1)	3.1 (0.3)	3.4 (0.1)	3.7 (0.1)
median	3.9	4.4	3.3	3.4

Results for 8 specimen, mean peak pressure of 5 measuring cycles of talonavicular joint (TN) and calcaneocuboid joint (CC), for native situation vs. ankle fusion

### Effects of tibiotalar arthrodesis on the talonavicular joint

We observed a varied increase in intraarticular force and peak pressure in the talonavicular joint following arthrodesis. The median force changed from a native value of 92 N (95% CI: 64 N–145 N) to 100 N (95% CI: 69 N–164 N) upon arthrodesis. Over all feet, the increase in force on the talonavicular joint was statistically significant (p = 0.027), while we also saw a significant increase in the peak pressure (p = 0.0005) from native 3.9 MPa (95% CI: 3.1 MPa–5.7 MPa) to 4.4 MPa (95% CI: 3.5 MPa–6.2 MPa).

### Effects of tibiotalar arthrodesis on the calcaneocuboid joint

In the calcaneocuboid joint, we recorded lower values for force upon arthrodeses, the effect was statistically significant (p = 0.029). We observed a median decrease from the native load, from 54 N (95% CI: 28 N–91 N) to 48 N (95% CI: 24 N–82 N) upon arthrodesis; regarding intraarticular peak pressure, we saw a change from native 3.3 MPa (95% CI: 2.7 MPa–4.4 MPa) to 3.4 MPa (95% CI: 2.6 MPa–4.2 MPa). This effect was not significant (p = 0.098).

### Change in stress distribution between talonavicular and calcaneocuboid joints

Our measurements indicate a tendency of relocation of the force onto the talonavicular joint after arthrodesis with a reduction of the force on the calcaneocuboid joint. Overall, this relocation of force is statistically not significant (p-value of interaction: 0.30). The effect of the relocation of maximum peak pressure onto the talonavicular joint is also not detectable with statistical significance (p = 0.11).

In our experiment, there is a trend correlating the size of the foot preparations with the imparted force, where larger preparations in the model produced higher values than smaller preparations regarding the sum of the two parts of the Chopart joint (p = 0.0043). There is no statistically significant correlation of peak pressure with foot size (p = 0.07).

## Discussion

The transfer of force from the tibia to the ground when walking is imparted through an extension/flexion movement of the foot against the lower leg and also an eversion/inversion movement in the hindfoot and an abduction/adduction movement in the forefoot. Previous examinations show here that about 4/5 of the extension/flexion movement is imparted through the ankle joint and 1/5 through the Chopart joint [9]. The latter can greatly increase the extent of movement in the case of arthrodesis in individual cases; this is associated with greater stress on the joint [3,11]. The mobility of the foot, in terms of dorsiflexion and plantar flexion, is given at about 10° for each following tibiotalar arthrodesis, which has been described as sufficient for normal walking [12]. Furthermore, a change in the movement axis between foot and lower leg in the stance phase due to arthrodesis was demonstrated, with a greater than 100% increase in tibia rotation and hindfoot inversion and eversion [12,13]. This observation explains the increased stress in the medial column of the foot that we measured in our experiments. Due to the increased calcaneal eversion in dorsiflexion, the foot rolls – in the case of lacking mobility – off over the medial column, especially since a forceful push-off position in a plantar flexion position can no longer be naturally achieved in the case of a fused ankle joint.

The complex anatomy of the Chopart joint, with a ball-and-socket articulation at the medial column of the foot and a saddle articulation at the lateral column, is an expedient biomechanical construction for accomplishing the complex tasks of the foot, namely optimally adapting to uneven surfaces upon first heel contact and forcefully pushing the foot off the ground at the end of the walk cycle. The locking mechanism of the Chopart joint upon calcaneal inversion was described by Elftman 1960 [14]. This locking was attributed to divergence of the transverse axes of the talonavicular joint and the calcaneocuboid joint in the case of calcaneal inversion or plantar flexion. In contrast, the joint is loose upon eversion of the hindfoot or dorsal flexion caused by parallel alignment of the joint axes [15]. This anatomical feature allows optimal adaptation of the foot at the beginning of the rolling motion immediately after contact of the heel with the ground. We observed that this locking effect of the Chopart joint is supported by an active spreading-out movement of the dorsal, convex head of the talus and of the plantar convex part of the calcaneocuboid joint, measurable by a pressure increase dorsal in the talonavicular joint and plantar in the calcaneocuboid joint at the push-off phase [16] and it is easy to understand that this locking mechanism is compromised by the described biomechanical changes with a resulting insufficiency of the joint stabilisation during push-off. This effect may even provide more stress on the talonavicular joint.

Since it is not possible to make in-vivo measurements of intraarticular pressures or analyse the imparted forces in people, the biomechanical joint stresses must be simulated in a model. In this regard, computer simulations and cadaver experiments are possible approaches. Cadaver experiments are thus, even in the present study, only a model in the simulation of physiological processes and thus limitations of their meaning must be taken into account. An essential advantage of our experiment is the ability of the test apparatus to simulate even extrinsic tendon pull on the foot. As such, our simulation is a test setup that corresponds more closely to reality than previously published experiments. The inability of earlier cadaver experiments to simulate this has been described as significantly disadvantageous [13].

We observed much interindividual variance among our preparations. This can be explained by the fact that the preparations were of different sizes, and a fluctuation of the results in the range of 100% can be reasonably explained by different individual anatomic conditions, such as body weight. We were able to make a statistical correlation between imparted force and foot size. Since we activated the same muscles in all preparations, we assume relatively too high measurements for the smaller feet and too low measurements for the larger feet. After averaging the results, we obtained a realistic average value.

One specific possibility for error in our experiments was the possibility that forces were being imparted off to the side of the pressure films, which cannot be ruled out with absolute certainty in the narrow joints, even after careful preparation and placement of the films.

Determining force and pressure conditions in the articulations of the hindfoot is technically difficult. In a previous study the loads on the subtalar joint and the talonavicular joint following tibiotalar arthrodesis were determined in a servohydraulic test machine [17]. The Fuji pressure films used were the same thickness as our Texscan films at 0.3 mm, thus in principle it must be taken into account that the film could become stuck in the joint; furthermore, unintentional slipping is also conceivable [18]. Also, the effect of the intrinsic foot musculature has not been factored into the model, but can be assumed to be minimally relevant in the examination of a hindfoot articulation. The reproducibility of the individual measurements in our test was predominantly very good. The potential error in observing the forces arises in the analysis of the native values; the error is eliminated, however, in the comparison of the native values with the arthrodesis.

## Conclusion

After tibiotalar arthrodesis, we measured inconsistent interindividual changes in the Chopart joint. The imparted force and, consequently, the peak pressure both rise on the medial column of the foot in the talonavicular joint and the force fall on the lateral column in the calcaneocuboid joint. When seen in connection with the clinically observed and scientifically described adaptation processes – with an increase in the degree of movement in the talonavicular joint after ankle joint arthrodesis, increased tibial rotation and increased calcaneal eversion – this biomechanical fact impressively explains the appearance of cartilage degeneration on the medial dorsum of the foot. The increase in peak pressure after tibiotalar arthrodesis at the point of highest loading during the forceful push-off phase of the foot must be particularly problematic. As a conclusion of our experiment, it would be desirable to reduce the force imparted on the medial column that results from arthrodesis, with lateral relocation of the imparted force. Further clinical investigations are required to test such an effect, say, by simple shoe adjustment with support of the lateral forefoot or a restriction of hindfoot eversion after tibiotalar arthrodesis, the objective being to improve the long-term results.

## Competing interests

The author(s) declare that they have no competing interests.

## Authors' contributions

AS: planning, writing and leading the study; TH: statistical analyses and support in mathematical preparation of the complex interpretation of data; OM: Preparation of the data writing MatLab programms to interpret raw data and creates figures; NW: organized financial support and constructed the testing apparatus

## Pre-publication history

The pre-publication history for this paper can be accessed here:


